# An evaluation of approaches for rare variant association analyses of binary traits in related samples

**DOI:** 10.1038/s41598-021-82547-z

**Published:** 2021-02-04

**Authors:** Ming-Huei Chen, Achilleas Pitsillides, Qiong Yang

**Affiliations:** 1 National Heart, Lung and Blood Institute’s Framingham Heart Study, Population Sciences Branch, Division of Intramural Research, National Heart, Lung and Blood Institute, Framingham, MA 01702 USA; 2 Department of Biostatistics, Boston University School of Public Health, Boston, MA 02118 USA

**Keywords:** Population genetics, Rare variants, Statistics

## Abstract

Recognizing that family data provide unique advantage of identifying rare risk variants in genetic association studies, many cohorts with related samples have gone through whole genome sequencing in large initiatives such as the NHLBI Trans-Omics for Precision Medicine (TOPMed) program. Analyzing rare variants poses challenges for binary traits in that some genotype categories may have few or no observed events, causing bias and inflation in commonly used methods. Several methods have recently been proposed to better handle rare variants while accounting for family relationship, but their performances have not been thoroughly evaluated together. Here we compare several existing approaches including SAIGE but not limited to related samples using simulations based on the Framingham Heart Study samples and genotype data from Illumina HumanExome BeadChip where rare variants are the majority. We found that logistic regression with likelihood ratio test applied to related samples was the only approach that did not have inflated type I error rates in both single variant test (SVT) and gene-based tests, followed by Firth logistic regression that had inflation in its direction insensitive gene-based test at prevalence 0.01 only, applied to either related or unrelated samples, though theoretically logistic regression and Firth logistic regression do not account for relatedness in samples. SAIGE had inflation in SVT at prevalence 0.1 or lower and the inflation was eliminated with a minor allele count filter of 5. As for power, there was no approach that outperformed others consistently among all single variant tests and gene-based tests.

## Introduction

Family data provide a distinct advantage of detecting rare risk genetic variants associated with human traits. One of the possible reasons for lack of reproducibility in population-based genetic studies of rare variants is heterogeneity, that is, multiple causal rare variants are underlying the disease. Using family samples can increase the presence of the disease predisposing alleles thus has a better chance to detect the association than using unrelated samples.

With continuous traits, linear mixed effects model (LME) is the commonly used method for association analyses with related samples implemented in popular software packages such as GCTA^1^, RAREMETALWORKER^2^, EPACTS^3^, FREGAT^4^ (Family REGional Association Tests) and seqMeta (https://cran.r-project.org/web/packages/seqMeta/index.html). These software packages use either a relationship coefficient matrix based on pedigree information or a genetic/genomic relationship matrix (GRM) estimated from genotype data to account for relatedness. Those that use a GRM (e.g. GCTA, RAREMETALWORKER, EPACTS) can additionally account for population stratification.

For binary traits, the field is more complex possibly due to the need to deal with computational and separation issues caused by low frequencies in some genotype category and/or event outcome. The aforementioned packages either handle an unrelated subset only, which results in power loss, or use LME and treat binary traits as continuous for fast computing speed, which however produces uninterpretable effect estimate.

We have developed RVFam that provides tools for analyzing three types of traits: continuous, binary and survival measured in family samples^5^. For binary outcomes, RVFam fits a generalized linear mixed effects model (GLMM). Unlike LME, there is no close form solution to the maximum likelihood of GLMM, therefore binary trait association analyses using RVFam/GLMM take more time than using LME.

Additional tools for binary outcomes have emerged in recent years that tried to address the computational and separation issues caused by low frequencies in some genotype category and/or event outcome. GMMAT (generalized linear mixed model association test) fits GLMM with a GRM and derives a score statistic for association analyses which is faster than the maximum likelihood method^6^. However, such score statistic can be inflated when applied to rare variants. Recently Zhou et al.^7^ proposed Scalable and Accurate Implementation of Generalized mixed model (SAIGE) that uses the saddle point approximation to calibrate the distribution of score test statistics to better handle extremely unbalanced data. It reduces computational and memory cost by utilizing strategies such as the average information restricted maximum likelihood algorithm and replacing the spectral decomposition with the preconditioned conjugate gradient method to solve linear systems and avoid the need of inversing GRM.

Firth logistic regression^8,9^ was proposed to resolve the separation issue by adding a penalty for sparsity on the likelihood of a logistic regression. It has been shown that Firth logistic regression maintained a valid type I error when applied to rare variants analyses in unrelated samples^10^. Note Firth logistic regression does not account for relatedness and population structure. However, population structure can be controlled by adjusting for principal components of variance-standardized relationship matrix estimated using genome-wide data in association testing as is also the practice of other methods which we compared in this study. Here we are interested in examining its performance on both unrelated and family samples.

In this paper, we perform extensive simulation studies to compare RVFam, GMMAT, seqMeta, EPACTS-q.emmax (LME treating binary traits as continuous), logistic regression with likelihood ratio test (LRT), Firth logistic regressions and SAIGE. These approaches are population based as opposed to family based methods such as family based association tests (FBAT^11^), rare-variant transmission disequilibrium test (RV-TDT^12^), rare-variant generalized disequilibrium test (RV-GDT^13^), and a rare-variant association test for affected sibships^14^. The family based methods use within family information to control for population stratification and can be used for association analyses of binary phenotypes measured on families. However these family-based methods may be less powerful compared to population based methods when the latter are able to control for population stratification through covariate adjustment. In this study, we focus on the population based methods.

## Materials and methods

### Existing methods

There are few software packages available for testing association between rare variants and binary outcomes with family data. We selected RVFam, GMMAT and SAIGE designed for analyzing this type of data. We also compared software packages designed for analyzing rare variants with unrelated samples in the same simulations using a subset of unrelated samples from the family data—we selected seqMeta R package and logistf R package^9^ implementing Firth’s logistic regression method^8^. We also included logistic regression with LRT and EPACT’s q.emmax that uses LME and treats binary outcomes as continuous for comparison.

RVFam (https://cran.r-project.org/web/packages/RVFam/) is an R package/wrapper for rare variants analysis with family data that can be applied to continuous, binary or survival traits^5^. For binary traits, RVFam uses GLMM with a logistic link by calling the glmer() function in lme4 R package. Users can choose a gene-based test from either a collapsing burden test^15^, a Madsen and Browning’s burden test^16^ or a sum of squares (SSQ) test^17^ in addition to single variant test (SVT). RVFam reports p-values from LRT except for the SSQ test that is a chi-square test. When a convergence issue occurs in estimating the variance–covariance matrices of the random effects in the glmer() function call (page 16 of lme4’s reference manual, https://cran.r-project.org/web/packages/lme4/lme4.pdf), RVFam catches warning messages returned from lme4 and provides a remark. Even though the warning messages do not necessarily imply incorrect glmer fitting, the resulting regression coefficients may not be reliable.

GMMAT (Generalized Linear Mixed Model Association Tests, https://github.com/lin-lab/GMMAT) is an R package that uses a logistic mixed model with a GRM to account for population substructure and relatedness in genetic association studies of binary traits^6^. GMMAT also has a component for analyzing continuous traits. Gene-based tests including burden tests and sequence kernel association test^18^ (SKAT) are available in addition to SVT. GMMAT is the component for binary traits in GENESIS (GENetic EStimation and Inference in Structured samples, https://bioconductor.org/packages/release/bioc/html/GENESIS.html) R package available on the Analysis Commons, a cloud-based computing platform, for National Heart, Lung, and Blood Institute’s TOPMed project.

SAIGE, (Scalable and Accurate Implementation of GEneralized mixed model, https://github.com/weizhouUMICH/SAIGE), is a software implemented for efficiently controlling for unbalanced case–control ratios, sample relatedness and population stratification for genome-wide association study (GWAS) of large sample sizes. SAIGE uses saddle point approximation to control for case–control imbalance in a logistic mixed effects model and reports score test results^7^. Advanced optimization techniques are used in SAIGE and their efficiency is seen in its applications to UK Biobank data of 408,961 white British samples. Burden test and SKAT are also available in SAIGE.

seqMeta (https://cran.r-project.org/web/packages/seqMeta/index.html) is an R package that can be used for individual study analysis and meta-analysis, and it can be applied to continuous, binary and survival traits using unrelated samples, with exception of continuous traits that can be applied to family data using LME. For binary outcomes, seqMeta uses logistic regression. Burden tests and SKAT are implemented in addition to SVT.

Logistic regression with LRT is carried out using the glm () function in R and is denoted as GLM. Based on RVFam’s binary traits component framework, we implemented a function for SVT and gene-based tests using GLM. Firth logistic regression is proposed to correct asymptotic bias of maximum likelihood estimates and to provide a solution to separation by using a penalized likelihood function^8,9^. The approach is implemented in logistf R package (https://cran.r-project.org/web/packages/logistf). We created a function for gene-based tests and SVT using Firth logistic regression implemented in logistf R package based on RVFam’s binary traits component. Theoretically both GLM and Firth logistic regression (denoted Firth test) are only applicable to unrelated samples as they do not account for relatedness.

EMMAX (efficient mixed-model association eXpedited), is a component for analyzing continuous traits using LME in EPACTS^3^ (Efficient and Parallelizable Association Container Toolbox, https://genome.sph.umich.edu/wiki/EPACTS). Treating binary traits as continuous was seen in some GWAS literatures using popular software that implement LME^1,3,19–21^. Here we used q.emmax option in EPACTS and treated binary outcomes as continuous.

### Simulation designs

We conducted two simulation studies to evaluate the performance of aforementioned methods in terms of type I error rate and statistical power for testing genetic association.

In the first simulation study, we used the real genotypes of 246,670 Single Nucleotide Polymorphisms (SNPs) captured on the Illumina HumanExome BeadChip^22^ and simulated phenotypes of 3380 Framingham Heart Study (FHS) Offspring Cohort participants with exome chip data in 1147 pedigrees containing participants from three generation recruitments^23–25^. The exome chip data consists of 85% rare variants defined as variants with minor allele frequency (MAF) less than 0.01. Among the 3,380 samples, a subset of 1,868 unrelated samples were identified. We first simulated a QTL and its linked continuous trait using the FHS sample with SOLAR^26^. The QTL (MAF 0.05) explained 2% of the trait variance (genotypic mean decided by sqrt(0.02/(2 × 0.05 × 0.95)) under an additive model) and the polygenic variance proportion was set to be 25%. With prevalence (k = 0.01, 0.05, 0.1 and 0,2), genotype relative risk (GRR = 1.5) and the QTL allele frequency, we computed the probabilities of being affected per genotype under an additive model and used them as quantiles to dichotomize simulated continuous traits per genotype group to obtain our binary outcomes. To evaluate type I error rates, 100 replicates of phenotype data were generated and tested for association with all exome chip variants independent of the QTL. For power simulation, as RVFam has a survival component, we simulated a survival trait and took the event variable as our binary trait, so we could evaluate both survival (not included in the present study) and binary components. The survival trait was simulated to follow a Weibull distribution with shape parameter 1 (constant failure rate over time) and scale parameter that incorporated normally distributed random effects and effects of the five selected independent (r^2^ < 0.0063) QTL with various MAF from the ABO gene that contained 36 variants (4 monomorphisms) in the exome chip data. The MAF, r^2^ and assigned effect information of the five QTL (exm792698, exm792721, exm792730, exm792750, and exm792745) are given in Supplementary Table S1. The random effects were simulated using the FHS sample with SOLAR, where polygenic variance was 25% (QTL variance was 0). The prevalence was 0.2. We simulated 100 replicates to assess statistical power. Both SVT and gene-based tests were conducted for all considered approaches. For gene-based tests, MAF ranges of (0, 0.01) and (0, 0.05) were used to select SNPs. We used T1/T5, SSQ1/SSQ5 and SKAT1/SKAT5 to denote the three gene-based tests with MAF range 0.01/0.05, Li and Leal 2008’s collapsing/burden test, Pan 2009’s SSQ test and SKAT, respectively. Note the burden test implemented in all methods use a variant weight of 1, SSQ and SKAT use a variant weight of beta(1,25). The Bonferroni corrected genome-wide significant threshold was set as 0.05/246,670 = 2.027E−7 and 0.05/26,651 = 1.876E−6 for SVT and gene-based tests, respectively.

In the second simulation study, we were interested in evaluating the type I error rates of SVT using simulated genotypes and phenotypes based on the same FHS samples. We simulated dichotomized traits similarly as in the first study with two differences: (i) polygenic variance was additionally set to 50%, (ii) a SNP independent of the QTL was simulated to have MAF of 0.0001, 0.0005, 0.001, 0.005, 0.01, 0.05, 0.1 and 0.3, and was tested to estimate type I error rates. That is, we have polygenic variance (*vg*) at 0.25 and 0.5, prevalence (*k*) at 0.01, 0.05, 0.1 and 0.2, and MAF (*p*) at 0.0001, 0.0005, 0.001, 0.005, 0.01, 0.05, 0.1 and 0.3, 64 scenarios in total and 10,000 replicates for each scenario. Thus, each *vg* has 32 scenarios (4 *k*’s and 8 *p*’s), each *k* has 16 scenarios (2 *vg*’s and 8 *p*’s) and each *p* has 8 scenarios (2 *vg*’s and 4 *k*’s). Type I error rates were evaluated at significance levels of, 0.05, 0.001 and 5E−6. An inflated type I error rate was claimed when a type I error rate estimate was greater than significance level inferred by a one-sided proportion test.

In both simulation studies, all methods including GLM and Firth test were applied to related samples, except for seqMeta. Firth test was also applied to unrelated samples. All analyses and figures were conducted or produced using the R language and environment^27^.

## Results

### First simulation study

Table 1 presents the type I error rate results. Type I error rate was computed by total number of SNPs/genes that passed genome-wide significance threshold in 100 replicates divided by total number of SNPs/genes in 100 replicates, which has been calibrated by dividing the significance threshold. A value greater than 1 in Table 1 indicates inflation.

**Table 1:** Calibrated type I error rate (Type I error rate was computed by total number of SNPs/genes that passed genome-wide significance threshold in 100 replicates divided by total SNPs/genes in 100 replicates, which has been calibrated by dividing the significance threshold. A value greater than 1 indicates inflation) of the first simulation study.

Prevalence	RVFam					GMMAT					Firth				
	SVT	T1	SSQ1	T5	SSQ5	SVT	T1	SKAT1	T5	SKAT5	SVT	T1	SSQ1	T5	SSQ5
0.01	11,478.2	2138.8	11,478.2	2138.8	4550.8	7618.4	843	1582	2431	2294.8	0.4	0.2	109.8	0.2	100.4
0.05	0.4	0.8	0.4	0.8	0.4	937.6	154.4	266.6	121.8	201.4	0.6	0.6	0.6	0.2	0.6
0.1	0.4	0.2	0.4	0.2	0	127.6	31.2	46.4	23.2	34.4	0.6	0.8	0.2	0.6	0.2
0.2	0	0.4	0	0.4	0.2	2.2	2	3.2	1.6	2.8	0	0.2	0	0.2	0

Prevalence	q.emmax					seqMeta (unrelated)					Firth (unrelated)				
	SVT	T1	SKAT1	T5	SKAT5	SVT	T1	SKAT1	T5	SKAT5	SVT	T1	SSQ1	T5	SSQ5
0.01	7923.2	1536.6	3447.8	1258.2	2611.8	19,126.4	2242.6	5155.6	1774.8	3752.4	0.2	0.8	242	0.8	225
0.05	562.8	195.4	332.2	159.6	257.2	1487.6	342	566.2	266.6	422.6	0.4	1	0.4	0.6	0.6
0.1	182.8	37	53.4	29.4	41.2	138.2	50.6	75.2	40.8	56.2	0	0.2	0	0.4	0
0.2	2.6	1.8	4	1.8	3.2	1.4	3	3.2	2.4	3	0	0.2	0	0.6	0

Prevalence	SAIGE					GLM									
	SVT	T1	SKAT1	T5	SKAT5	SVT	T1	SSQ1	T5	SSQ5					
0.01	1489.4	0	0.2	0	0.2	0	0	0	0	0					
0.05	6109.2	0.4	0.6	0.2	0.6	0.4	0.4	0.4	0.2	0.4					
0.1	1021.8	0.2	0	0	0.2	0.4	0.2	0	0.2	0					
0.2	0	0.4	0.6	0.2	0.8	0	0.4	0.2	0.4	0.2					

Genome-wide significance level for single variant tests (SVT) = 2.027E−7; for gene-based tests = 1.876E−6. Methods with “unrelated” are those applied to unrelated samples, all other approaches were applied to related samples. T1/T5, SSQ1/SSQ5 and SKAT1/SKAT5 denote the three gene-based tests with MAF threshold 0.01/0.05, collapsing/burden test, sum of squares test and SKAT, respectively.

GLM had the best performance on controlling type I error rates in both SVT and gene-based tests as no inflation was observed, followed by Firth tests that had slight inflation observed only in SSQ1 and SSQ5 at prevalence 0.01, SAIGE that had inflation only in SVT at prevalence 0.1 or lower, and then RVFam that had inflation in both SVT and gene-based tests only at prevalence 0.01. GMMAT, q.emmax and SeqMeta all had inflation across all scenarios in both SVT and gene-based tests. In general, type I error rates increased when prevalence decreased for methods with inflations. It was interesting that no inflation was also observed in GLM and Firth test applied to related samples and that SSQ tests applied to related samples had less inflation than applied to unrelated samples in Firth test.

**Figure 1:**
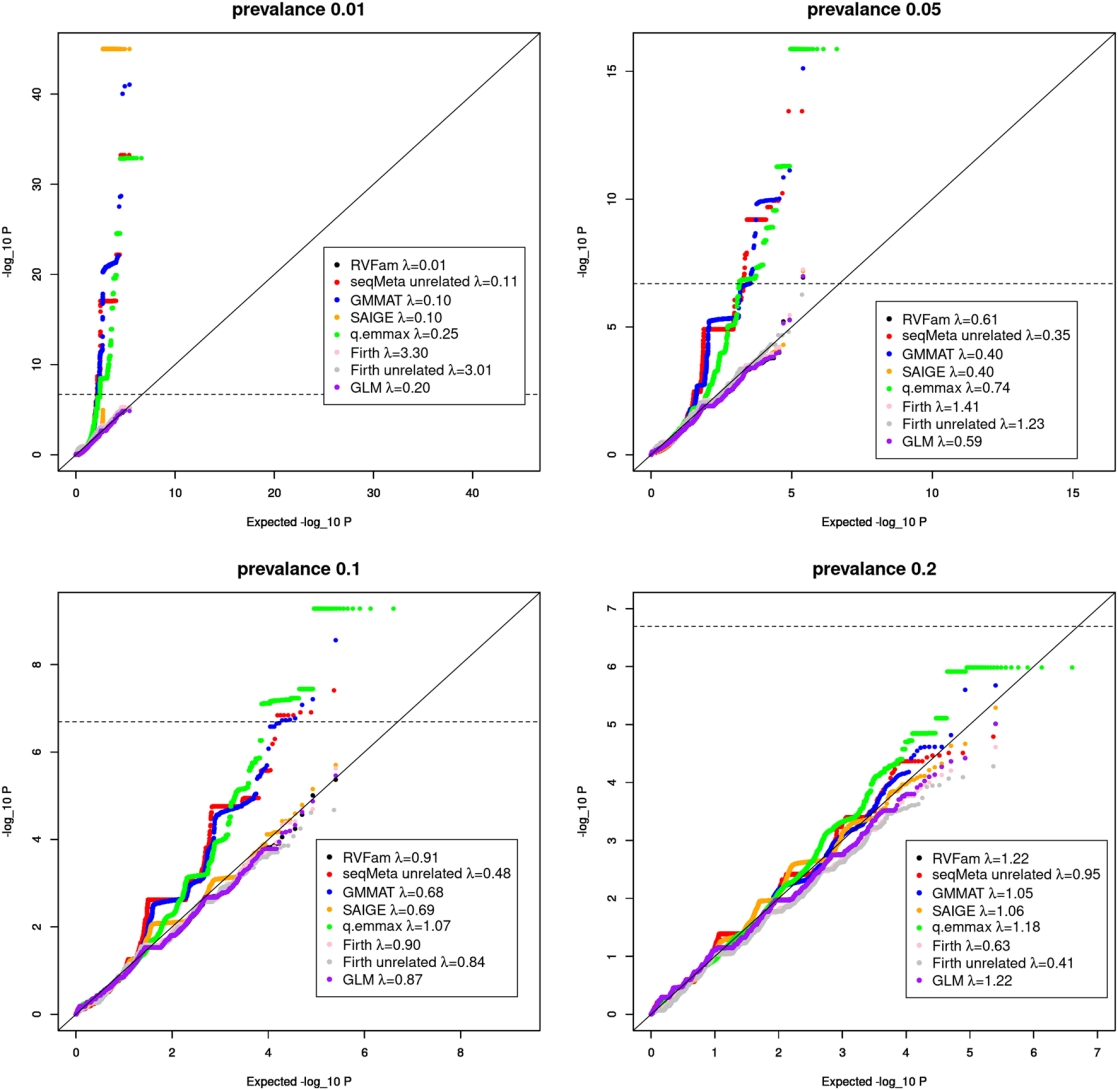
QQ plots of replicate 1 for assessing Type I error inflation in single variant tests (SVT) of the first simulation study.

As the majority of the exome chip data are rare variants, we applied two minor allele account (MAC) filters to exclude rare variants with MAC not greater than 5 and 10 to see whether this could eliminate the inflated type I error rates in SVT. Table 2 shows that inflation was eliminated for SAIGE only but remained for other approaches with inflated type I error rates. We generated QQ plots (Fig. 1) arbitrarily using results of the first replicate simulated data for evaluating type I error rates at each prevalence (0.01, 0.05, 0.1 and 0.2). Genomic inflation factor^28^ (λ) estimates were presented in the legend of each QQ plot. Dash line represents the genome-wide significance level for SVT, points above this line represent false positives. SeqMeta, GMMAT and q.emmax identified many false positives at prevalence 0.01, 0.05 and 0.1. SAIGE identified several rare variant (MAC < 3) false signals at prevalence 0.01 with very small p-values that were replaced by 1E-45 for better presentation. At prevalence 0.05, GLM, RVFam, Firth test and SAIGE identified one false positive each. No false positive was detected at prevalence 0.2. Firth (unrelated) test did not identify any false positive. The results agreed with Table 1 in general. In terms of λ, interestingly, the estimates of Firth tests’ decreased from > 3 to < 0.64 as prevalence increased from 0.01 to 0.2, while the trend was opposite for other approaches.

**Table 2:** Estimated type I error rates (Type I error rate was computed by total number of SNPs that passed genome-wide significance threshold in 100 replicates divided by total SNPs in 100 replicates, which has been calibrated by dividing the significance threshold. A value greater than 1 indicates inflation) after applying MAC filters of 5 and 10 in single variant tests (SVT) of the first simulation study.

Prevalence	RVFam		GMMAT		Firth		q.emmax		seqMeta (unrelated)		Firth (unrelated)		SAIGE		GLM	
	MAC5	MAC10	MAC5	MAC10	MAC5	MAC10	MAC5	MAC10	MAC5	MAC10	MAC5	MAC10	MAC5	MAC10	MAC5	MAC10
0.01	9560.2	8219.6	675.8	296	0.2	0.2	1912.6	828.8	5863.2	1561.8	0	0	0	0	0	0
0.05	0.4	0.2	213.6	44.6	0.6	0.4	274.8	56.4	545.2	157.6	0.4	0.2	0.4	0.2	0.4	0.2
0.1	0.4	0.4	45.4	11.8	0.6	0.6	61	14.2	95	30.6	0	0	0.4	0.4	0.2	0.2
0.2	0	0	2.2	1.4	0	0	2.6	1.4	1.4	1.2	0	0	0	0	0	0

Genome-wide significance level: SVT = 2.027E−7. Methods with “unrelated” are those applied to unrelated samples, all other approaches were applied to related samples.

**Figure 2:**
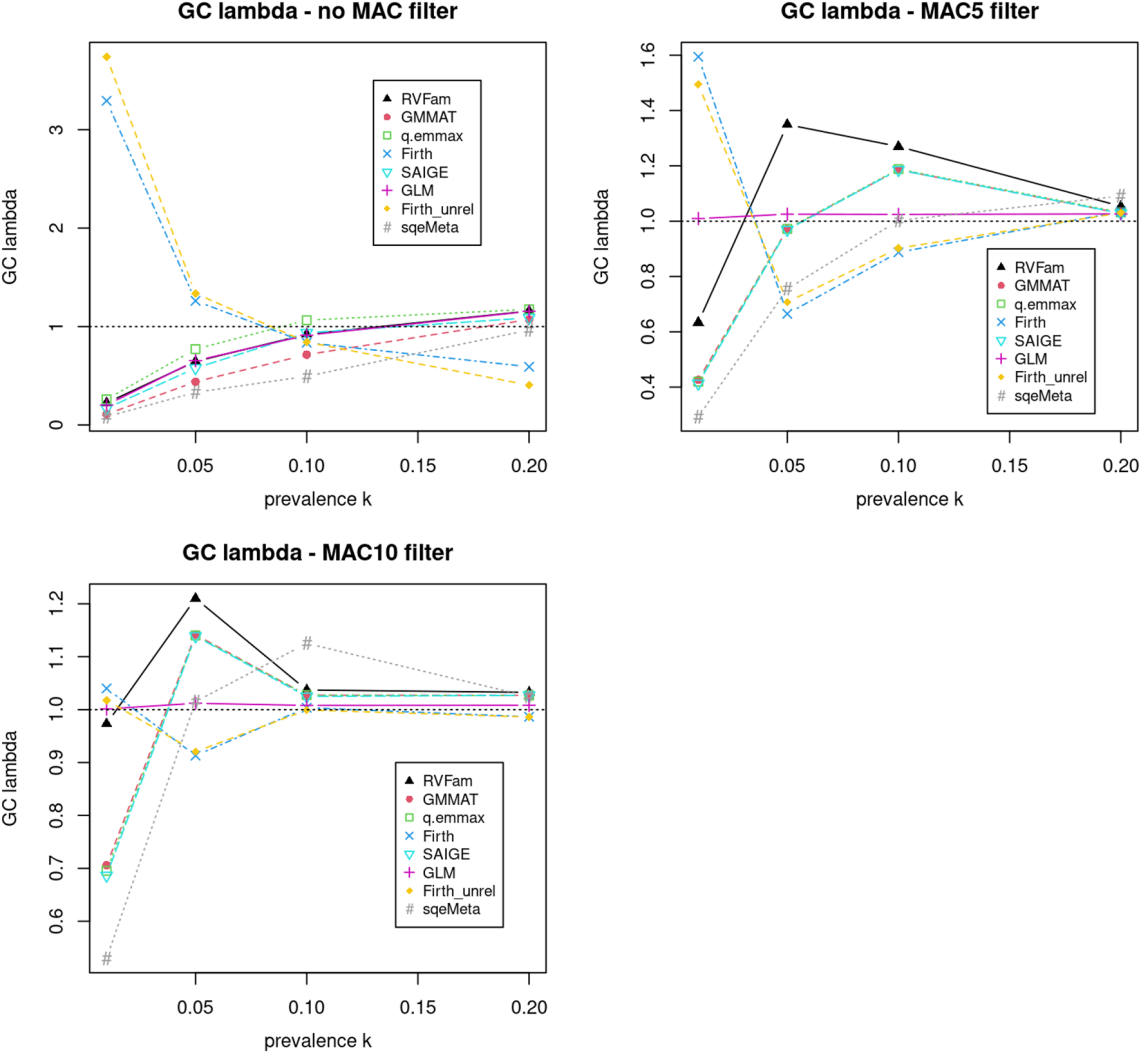
Plot of average genomic control parameter (λ) estimate from 100 replicates versus prevalence *k* in the first simulation study for evaluating type I error rates in SVT without minor allele count (MAC) filter (top left), with MAC5 filter (top right) and with MAC10 filter (bottom left).

We further computed λ in SVT of all replicates at each prevalence for each approach and then made average λ versus prevalence plots (Fig. 2). When no MAC filter was applied (top left panel), the plot essentially presents λ estimates similar to what were reported in Fig. 1 legends. The genomic inflation factor λ evaluates the medium of the test statistic distribution compared to that of the expected under the null hypothesis of no association. It is relevant because for example in meta analyses where moderate test statistic values from multiple studies are cumulated, and high λ values in some studies may result in inflation in overall summary statistics. The plots show Firth tests had opposite trend compared to others. At low prevalence (0.01 and 0.05) and no MAC filter, all approaches had low λ estimates except for Firth tests. In this case, genomic control correction^28^ may need to be applied to correct for inflation observed in Firth tests, which may result in a lower power for variants with p-values at the tail of the distribution. As rare variants are the dominant majority in exome chip data, one may apply the correction to rare variants only. When either MAC filter was applied, unlike other approaches that had fluctuating λ estimates at different prevalences, GLM had stable λ estimates very close to 1.

Table 3 presents the power results. Obviously approaches applied to the whole 3,380 samples had better power than approaches applied to the unrelated subset, and gene-based tests had better power than SVT for the rare variants. T1 had better power than T5 because three rare QTL of the same effect direction were selected in T1 and T5 further included a QTL with different effect direction that weakened the total effect and the association of ABO gene. As SSQ and SKAT tests are effect direction insensitive, we expect SSQ5/SKAT5 to have better power than SSQ1/SKAT1, which can be seen in GLM, RVFam, SAIGE and Firth tests, however, not in other approaches. There was no approach that consistently outperformed others in all scenarios. We particularly compared GLM, SAIGE and Firth test applied to related samples. For SVT, GLM and Firth test had better power in identifying the common QTL exm792745, while SAIGE had slightly better power in detecting the rare variant exm792721. For gene-based tests, GLM’s SSQ1/5 had better power in detecting ABO gene than Firth test and SAIGE, while Firth test and SAIGE had slightly better power in T1 than GLM.

**Table 3:** Power analysis results of the first simulation study.

Method	SVT power (%)					Gene-based test power (%)			
	exm792698	exm792721	exm792730	exm792745	exm792750	T1	SSQ1/SKAT1	T5	SSQ5/SKAT5
**RVFam**	**0**	**0**	**10**	**71**	**1**	**33**	**38**	**0**	**66**
GMMAT	0	12	32	63	0	57	60	0	39
q.emmax	0	20	32	63	0	59	65	0	38
**Firth (unrelated)**	**0**	**0**	**1**	**15**	**0**	**9**	**11**	**0**	**19**
**Firth**	**0**	**0**	**13**	**72**	**1**	**37**	**29**	**0**	**55**
seqMeta (unrelated)	0	20	5	10	0	24	28	0	3
**SAIGE**	**0**	**3**	**12**	**61**	**0**	**37**	**37**	**0**	**38**
**GLM**	**0**	**0**	**12**	**72**	**1**	**35**	**41**	**0**	**68**

Methods in bold are those not having severe inflation in type I error study and thus whose power can be compared together.

Genome-wide significance level for single variant tests (SVT) = 2.027E−7; for Gene-based tests = 1.876E−6. Methods with “unrelated” are those applied to unrelated samples, all other approaches were applied to related samples. T1/T5, SSQ1/SSQ5 and SKAT1/SKAT5 denote the three gene-based tests with MAF threshold 0.01/0.05, collapsing/burden test, sum of squares test and SKAT, respectively.

**Figure 3:**
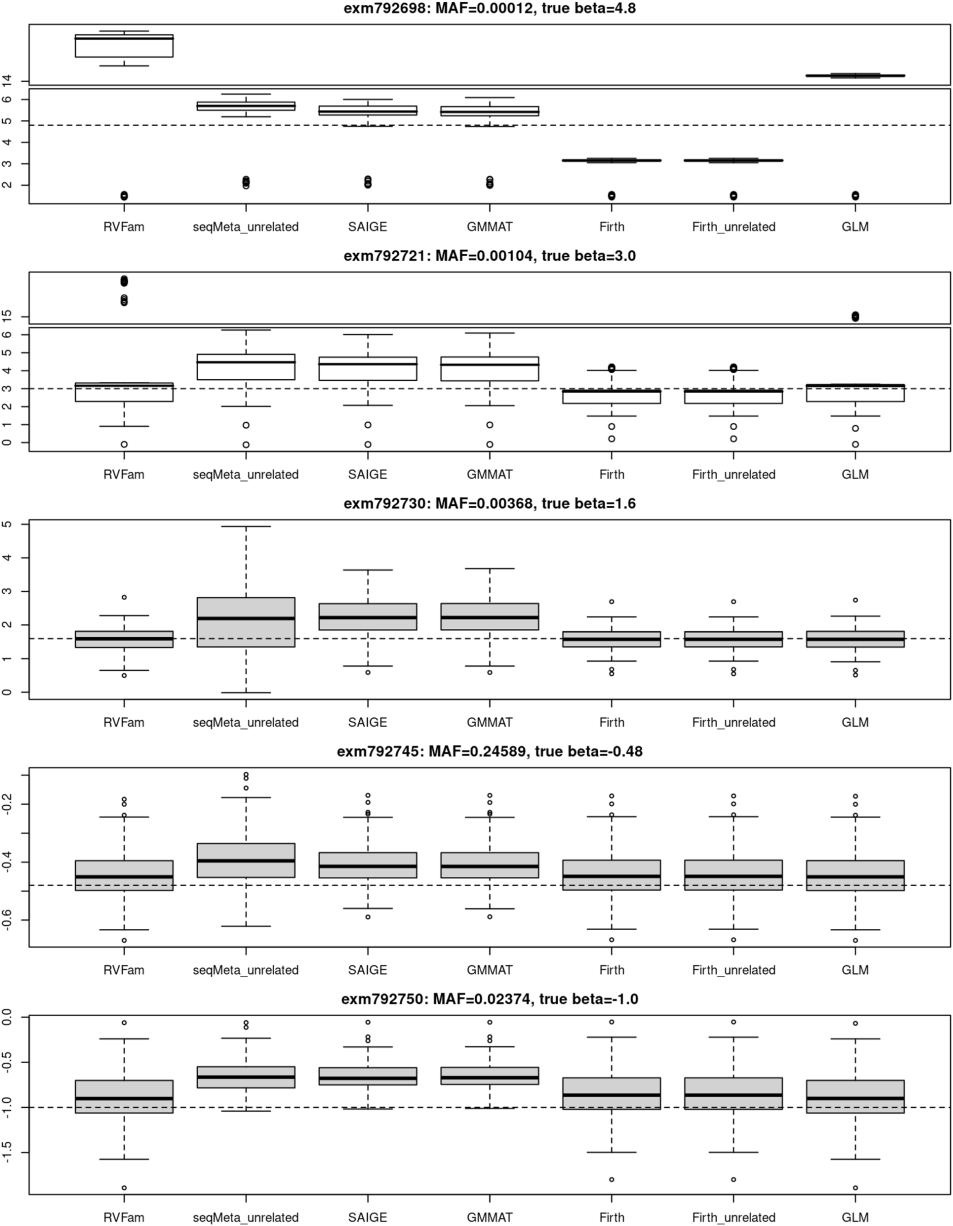
Box plots of beta estimates of the five selected QTL from power analysis of the first simulation study.

Figure 3 presents boxplots of regression coefficient (beta) estimates of the five QTL from all methods (except for q.emmax) and Supplementary Table S2 presents their averages and standard deviations. For RVFam in Supplementary Table S2, we reported results of all 100 replicates (4^th^ column) and results of replicates without convergence remark (5^th^ column). The convergence issues occurred to rare variants exm792721 and exm792698 and that led to very biased beta estimates. Excluding results with convergence issues, the biases were reduced (5^th^ column). First of all, Firth test applied to related samples consistently had the best precision (smallest standard deviation of effect estimates). For exm792698 (MAF 0.00012, true beta 4.8), GMMAT gave the closest estimate, followed by seqMeta, Firth tests, GLM and RVFam. For exm792721 (MAF 0.00104, true beta 3.0), Firth tests gave the closest estimate, followed by GMMAT, seqMeta, GLM and RVFam. While for the other three QTL, RVFam’s were closest to the true beta values, followed by GLM, Firth tests, seqMeta, GMMAT and SAIGE. Estimates from GMMAT and SAIGE were quite similar. Without excluding results with convergence issues, estimates from RVFam and GLM were also similar.

We also compared computing efficiency for approaches applied to related samples using a Linux Centos 6.6 cluster with a 2 ten-core 2.6 GHz Intel Xeon E5-2660v3 processor and 256 Gigabytes memory. We used a replicate of simulated data with prevalence 0.2 used in assessing type I error rates in the first simulation study and analyzed chromosome 1 only for SVT and gene-based tests with MAF threshold of 0.05. With a single thread, GLM, Firth test, SAIGE, GMMAT and RVFam took 9.13, 21.15, 15.6, 18.12, and 52.1 minutes.

Even though GLM outperformed other methods in maintaining correct type I error rates and computation efficiency, its biased effect estimate in rare variants can lead to biased results in the popular fixed effect meta-analysis. As Firth test was proposed to correct bias, we sought to replace GLM with Firth test in SVT for variants with some MAC threshold. We first computed mean of the effect estimate and its standard deviation of GLM and Firth test applied to related samples with MAC thresholds of 5 and 10 (Supplementary Table S3) using all replicates from the first simulation study evaluating type I error rates (where true effect was zero). We wanted to find a MAC threshold so that the GLM estimates of variants with MAC greater than the threshold were reasonably close to Firth test’s. The results showed that variants with MAC > 10 still showed differences between GLM and Firth test. We then tried MAC filters in disease samples (MAC.d) of 1, 2, and 3. The means were much closer to 0 and the standard deviations were smaller. We found MAC.d filter of 2 would be a good threshold as the means from GLM were even closer to 0 than from Firth test and the standard deviations were reasonably close to Firth test’s. The hybrid strategy that uses GLM for variants with MAC.d > 2 and Firth test otherwise, significantly reduced bias in effect estimation while keeping correct type I error rates and similar power in SVT.

### Second simulation study

Table 4 summarizes the results of the second simulation study. Numbers in the table indicate the number of scenarios with inflated type I error rates, i.e. false positive rates greater than level of significance inferred by a one-sided test. At significance level 0.05 (Table 4, α_1_), unlike the first simulation study where inflation increased when prevalence decreased in most approaches (except for Firth tests that had no inflation), no such trend was observed in all methods. No inflation was seen at MAF 0.0001 in all methods. RVFam and GLM had the same number of scenarios with inflation and did not have inflation at MAF ≤ 0.001, Firth test applied to unrelated samples did not have inflation at MAF ≤ 0.005. Firth test applied to unrelated samples, q.emmax and SAIGE had the least scenarios with inflation, while RVFam and GLM had the most scenarios with inflation (23/64).

**Table 4:** Type I error results of the second simulation study. Level of significance is 0.05 (α_1_), 0.001 (α_2_) and 5E-6 (α_3_).

	RVFam			GMMAT			SeqMeta (unrelated)			Firth			Firth (unrelated)			q.emmax			SAIGE			GLM		
	α_1_	α_2_	α_3_	α_1_	α_2_	α_3_	α_1_	α_2_	α_3_	α_1_	α_2_	α_3_	α_1_	α_2_	α_3_	α_1_	α_2_	α_3_	α_1_	α_2_	α_3_	α_1_	α_2_	α_3_
*vg* = 0.25	10	2	1	6	20	16	7	19	17	7	3	1	1	4	1	3	16	15	3	0	0	10	2	0
*vg* = 0.50	13	4	1	7	21	15	8	20	18	9	6	0	5	3	1	2	22	20	2	3	4	13	4	0
*k* = 0.01	1	1	2	2	13	12	3	14	12	3	4	1	2	4	1	2	12	12	2	0	0	1	1	0
*k* = 0.05	4	0	0	4	10	11	5	11	10	1	3	0	0	3	0	0	8	10	0	1	1	4	0	0
*k* = 0.1	9	1	0	1	10	7	0	8	12	5	0	0	1	0	1	1	10	9	1	0	0	9	1	0
*k* = 0.2	9	4	0	6	8	1	7	6	1	7	2	0	3	0	0	2	8	4	2	2	3	9	4	0
*p* = 0.0001	0	0	0	0	6	5	0	4	6	0	2	0	0	1	0	0	3	5	0	0	0	0	0	0
*p* = 0.0005	0	0	0	4	8	6	4	7	6	1	3	0	0	2	0	0	8	5	0	0	0	0	0	0
*p* = 0.001	0	0	0	4	8	7	4	8	7	2	2	0	0	3	0	2	8	7	2	0	0	0	0	0
*p* = 0.005	6	2	0	0	8	6	1	8	6	1	1	1	0	1	0	0	7	7	0	0	0	6	2	0
*p* = 0.01	6	1	2	0	6	4	3	7	6	1	0	0	4	0	0	0	7	5	0	0	2	6	1	0
*p* = 0.05	3	0	0	2	2	2	0	2	2	4	0	0	0	0	0	0	3	5	0	2	1	3	0	0
*p* = 0.1	4	2	0	1	2	1	1	3	1	3	0	0	1	0	1	2	1	1	2	1	1	4	2	0
*p* = 0.3	4	1	0	2	1	0	2	0	1	4	1	0	1	0	1	1	1	0	1	0	0	4	1	0

Numbers in the table indicate the number of scenarios with inflated type I error rates, i.e. false positive rates greater than level of significance inferred by a one-sided test. Methods with “unrelated” are those applied to unrelated samples, all other approaches were applied to related samples. We considered polygenic variance (*vg*) at 0.25 and 0.5, prevalence (*k*) at 0.01, 0.05, 0.1 and 0.2, and MAF (*p*) at 0.0001, 0.0005, 0.001, 0.005, 0.01, 0.05, 0.1 and 0.3, 64 scenarios in total and 10,000 replicates for each scenario. Thus, each *vg* has 32 scenarios (4 k’s and 8 *p*’s), each *k* has 16 scenarios (2 *vg*’s and 8 *p*’s) and each *p* has 8 scenarios (2 *vg*’s and 4 k’s).

At significance level 0.001 (Table 4, α_2_), the observation of inflation increased when prevalence decreased was seen in GMMAT, seqMeta and Firth test applied to unrelated samples. RVFam, GLM, both Firth tests and SAIGE had better control on type I error rates compared to GMMAT, seqMeta and q.emmax. RVFam and GLM again had the same number of scenarios with inflation.

At significance level 5E−6 (Bonferroni correction, Table 4, α_3_), GLM was the only approach that had no scenario with inflation. In general, more scenarios with inflation were identified when prevalence decreased (as observed in the first simulation study). The observation contradicted results in Table 4 at significance level of 0.05 (α_1_). Again, RVFam, both Firth tests and SAIGE had better control on type I error rates compared to GMMAT, seqMeta and q.emmax.

Overall, more scenarios with inflation were seen at polygenic variance 50% compared to polygenic variance 25%, no matter which significance level was used. GMMAT, seqMeta and q.emmax had more scenarios with inflation compared to others at α_2_ and α_3_, while RVFam, GLM and Firth test applied to related samples had less scenarios with inflation when significance level was more stringent. The number of scenarios with inflation for SAIGE and Firth test applied to unrelated samples seemed stable and less than other approaches’, except for GLM at α3 that had no scenario with inflation.

## Discussion

In this paper we presented results of two simulation studies that compared several existing methods for rare variants association analysis of binary outcomes with family data. The first simulation study used exome chip data and simulated binary outcomes to mimic a whole genome study where rare variants are the dominant majority. GLM is the only approach that had no inflated type I error rates for both SVT and gene-based tests. Firth tests applied to either related or unrelated samples had inflated type I error rates only for SSQ1/5 at prevalence 0.01. Firth test is also implemented in EPACTS, however no gene-based Firth test has been implemented. We also applied Firth test in EPACTS for SVT of the first simulation study using unrelated samples, the results are quite similar to ours. SAIGE had inflation only in SVT but the inflation was eliminated with a MAC filter of 5.

RVFam reports a remark when lme4 generates convergence warnings (in estimating variance–covariance matrices of random effects) that are not necessarily due to incorrect fitting. However, Fig. 3 and Supplementary Table S2 show that convergence failures lead to biased beta estimates for the two rarest QTL in our power analysis. In addition, lme4’s reference manual also reports that the warnings tend to occur with large datasets (number of observations greater than 100,000 approximately). When a genome-wide significant signal is identified with a remark by RVFam, we suggest users follow necessary steps recommended by lme4 authors/maintainers to evaluate and resolve the warnings. One can also use GLM or Firth test to check given that both methods had the correct type I error rates in our simulations. In the two simulation studies for evaluating type I error rates, RVFam results were assessed without excluding those with a remark. If we exclude those with a remark, the type I error rates will improve. While in the power simulation, power was evaluated with remark being ignored (Table 3). However, this did not impact the estimated power, as the warnings occurred mostly in the two rarest QTL with 0 power to detect them. In Supplementary Table S2, we presented results with or without excluding variants with a remark for contrast. Excluding those with a remark did bring the regression coefficient estimates closer to the true values for the two rarest QTL.

The second simulation study demonstrates that for SVT at Bonferroni correction threshold, GLM did not identify any false positives and thus had 0 scenario with inflation, followed by Firth test applied to related samples (1 scenario with inflation), Firth test applied to unrelated samples and RVFam (each with 2 scenarios with inflation) and then SAIGE (4 scenarios with inflation).

Based on our simulation results, we recommend a hybrid strategy that uses GLM for gene-based tests and variants with MAC.d > 2 and Firth test otherwise in SVT for rare variant association analyses of binary outcomes with unrelated samples or family data. This strategy provided correct type I error rates, least biased effect estimation, good power and computation efficiency without necessity of filtering out variants. Even though theoretically GLM and Firth test do not account for relatedness in samples, our simulation results showed that they outperformed other approaches like RVFam, GMMAT and SAIGE that account for relatedness in controlling type I error rates when applied to related samples. We think this may be due to (i) the use of LRT, (ii) the fact that the familial correlation of complex binary outcomes is weaker than continuous traits’, and (iii) an appropriate genome-wide significance is used. In the case of population stratification, one can adjust for genetic principal components in association testing.

Our study evaluates the statistical performance of approaches applied to binary trait GWAS with family data using exome chip data from the FHS. We believe our results can be generalized to similar datasets. However there are limitations. First, we considered only population based methods that do not adjust for ascertainments, so they may be subject to ascertainment bias if the families were collected from proband(s) instead of randomly sampled, e.g. in cohort studies where sample collection does not depend on the disease status of other family members. Second, there are scenarios that were not considered in the study, e.g. rarer diseases (prevalence less than 0.01). So we could not assess e.g. whether GLM still have correct type I error rates for rarer diseases that can be considered for much larger sample sizes. In addition, it’s also likely that SAIGE and the hybrid strategy will need a larger MAC/MAC.d filter to avoid false positives.

## Supplementary Information

Supplementary Information.
