# Supplementary material for: An evaluation of approaches for rare variant association analyses of binary traits in related samples
Source: Sci Rep. 2021 Feb 4;11:3145. doi: 10.1038/s41598-021-82547-z (PMC7862354; doi:10.1038/s41598-021-82547-z)
Supplement: Supplementary file 1 — Supplementary Information. [file 41598_2021_82547_MOESM1_ESM.docx]

**An evaluation of approaches for rare variant association analyses of binary traits in related samples**

Ming-Huei Chen^1*^, Achilleas Pitsillides^2^, and Qiong Yang^2^

1.National Heart, Lung and Blood Institute's Framingham Heart Study, Population Sciences Branch, Division of Intramural Research, National Heart, Lung and Blood Institute, Framingham, MA 01702, USA

2.Department of Biostatistics, Boston University School of Public Health, Boston, MA 02118, USA

*ming-huei.chen@nih.gov

**Supplementary Table S1**. Information of five disease loci selected for power simulations.

|  | | | Pairwise squared correlation | | | | |
| --- | --- | --- | --- | --- | --- | --- | --- |
| Disease locus | MAF | Effect | exm792698 | exm792721 | exm792730 | exm792745 | exm792750 |
| exm792698 | 0.00012 | 4.80 | 1 | 5.21E-07 | 1.85E-06 | 1.59E-04 | 1.18E-05 |
| exm792721 | 0.00104 | 3.00 | 5.21E-07 | 1 | 1.57E-05 | 1.04E-04 | 1.01E-04 |
| exm792730 | 0.00368 | 1.60 | 1.85E-06 | 1.57E-05 | 1 | 2.22E-04 | 3.29E-05 |
| exm792745 | 0.24589 | -0.48 | 1.59E-04 | 1.04E-04 | 2.22E-04 | 1 | 6.23E-03 |
| exm792750 | 0.02374 | -1.00 | 1.18E-05 | 1.01E-04 | 3.29E-05 | 6.23E-03 | 1 |

**Supplementary Table S2**. Average beta (standard deviation) estimates of five disease loci in power analysis. Methods with “unrelated” are those applied to unrelated samples, all other approaches were applied to related samples.

| Disease locus | MAF | true beta | RVfam | RVfam* | Firth | Firth (unrelated) | SeqMeta (unrelated) | GMMAT | SAIGE | GLM |
| --- | --- | --- | --- | --- | --- | --- | --- | --- | --- | --- |
| exm792698 | 0.00012 | 4.80 | 14.48 (3.88) | 1.50 (0.05)/8 | 3.03 (0.45) | 3.03 (0.46) | 5.44 (1.01) | 5.20 (0.96) | 5.22 (0.96) | 13.11 (3.44) |
| exm792721 | 0.00104 | 3.00 | 6.06 (6.13) | 3.68 (3.76)/82 | 2.84 (0.87) | 2.84 (0.88) | 4.30 (1.19) | 4.17 (1.13) | 4.19 (1.13) | 5.63 (5.40) |
| exm792730 | 0.00368 | 1.60 | 1.59 (0.37) | 1.59 (0.37)/100 | 1.58 (0.35) | 1.51 (0.59) | 2.15 (1.00) | 2.22 (0.56) | 2.22 (0.55) | 1.58 (0.36) |
| exm792745 | 0.24589 | -0.48 | -0.44 (0.09) | -0.44 (0.09)/99 | -0.44 (0.09) | -0.43 (0.12) | -0.39 (0.10) | -0.40 (0.08) | -0.40 (0.08) | -0.44 (0.09) |
| exm792750 | 0.02374 | -1.00 | -0.91 (0.31) | -0.91 (0.31)/100 | -0.87 (0.30) | -0.87 (0.34) | -0.65 (0.18) | -0.65 (0.16) | -0.65 (0.16) | -0.91 (0.31) |

*Based on results without warning message, number after / represents the number of replications without warning message

**Supplementary Table S3**. Average beta (standard deviation) estimates of GLM and Firth test applied to related samples from the first simulation study used for evaluating type I error rates. MAC is minor allele count and MAC.d is MAC in disease samples.

|  | k | MAC ≤ 5 | MAC > 5 | MAC ≤ 10 | MAC > 10 | MAC.d ≤ 1 | MAC.d > 1 | MAC.d ≤ 2 | MAC.d > 2 | MAC.d ≤ 3 | MAC.d > 3 |
| --- | --- | --- | --- | --- | --- | --- | --- | --- | --- | --- | --- |
|  |  | mean (sd) | mean (sd) | mean (sd) | mean (sd) | mean (sd) | mean (sd) | mean (sd) | mean (sd) | mean (sd) | mean (sd) |
| GLM | 0.01 | -9.88 (2.74) | -5.33 (6.32) | -10.01 (2.95) | -4.07 (6.15) | -9.83 (3.96) | 0.09 (0.52) | -9.52 (4.29) | 0.05 (0.38) | -9.34 (4.45) | 0.04 (0.34) |
|  | 0.05 | -8.82 (5.04) | -2.68 (5.24) | -8.65 (5.25) | -1.46 (4.17) | -8.46 (5.38) | 0.14 (0.69) | -7.94 (5.66) | 0.06 (0.35) | -7.71 (5.74) | 0.04 (0.27) |
|  | 0.1 | -7.88 (6.61) | -1.50 (4.18) | -7.44 (6.65) | -0.59 (2.72) | -7.65 (6.44) | 0.19 (0.99) | -6.94 (6.64) | 0.08 (0.43) | -6.65 (6.66) | 0.05 (0.29) |
|  | 0.2 | -5.51 (7.89) | -0.56 (2.65) | -4.96 (7.57) | -0.13 (1.25) | -5.96 (7.45) | 0.30 (1.51) | -5.04 (7.46) | 0.12 (0.73) | -4.69 (7.37) | 0.07 (0.40) |
| Firth | 0.01 | 3.10 (0.53) | 0.69 (0.91) | 2.90 (0.67) | 0.39 (0.72) | 2.36 (1.18) | 0.14 (0.51) | 2.31 (1.21) | 0.09 (0.38) | 2.27 (1.23) | 0.07 (0.34) |
|  | 0.05 | 1.57 (0.64) | 0.17 (0.57) | 1.40 (0.74) | 0.06 (0.48) | 1.16 (0.91) | 0.18 (0.52) | 1.14 (0.91) | 0.09 (0.35) | 1.12 (0.91) | 0.06 (0.28) |
|  | 0.1 | 0.97 (0.76) | 0.06 (0.52) | 0.84 (0.81) | 0.01 (0.43) | 0.68 (0.87) | 0.20 (0.54) | 0.69 (0.88) | 0.10 (0.37) | 0.68 (0.87) | 0.07 (0.28) |
|  | 0.2 | 0.47 (0.92) | 0.01 (0.48) | 0.40 (0.92) | -0.00 (0.35) | 0.24 (0.91) | 0.22 (0.56) | 0.31 (0.92) | 0.12 (0.41) | 0.32 (0.92) | 0.08 (0.31) |
